# Singleton Sequence Type 382, an Emerging Clonal Group of Listeria monocytogenes Associated with Three Multistate Outbreaks Linked to Contaminated Stone Fruit, Caramel Apples, and Leafy Green Salad

**DOI:** 10.1128/JCM.02140-16

**Published:** 2017-02-22

**Authors:** Yi Chen, Yan Luo, James Pettengill, Ruth Timme, David Melka, Matthew Doyle, Alikeh Jackson, Mickey Parish, Thomas S. Hammack, Marc W. Allard, Eric W. Brown, Errol A. Strain

**Affiliations:** Center for Food Safety and Applied Nutrition, Food and Drug Administration, College Park, Maryland, USA; University of Iowa College of Medicine

**Keywords:** outbreak, clone, singleton ST382, whole-genome sequencing

## Abstract

Three multistate outbreaks between 2014 and 2016, involving case patients in and outside the United States, were linked to stone fruit, caramel apples, and packaged leafy green salad contaminated with Listeria monocytogenes singleton sequence type 382 (ST382), a serotype IVb-v1 clone with limited genomic divergence. Isolates from these outbreaks and other ST382 isolates not associated with these outbreaks were analyzed by whole-genome sequencing (WGS) analysis. The primary differences among ST382 strains were single nucleotide polymorphisms (SNPs). WGS analysis differentiated ST382 from a clonal complex 1 outbreak strain co-contaminating the caramel apples. WGS clustered food, environmental, and clinical isolates within each outbreak, and also differentiated among the three outbreak strains and epidemiologically unrelated ST382 isolates, which were indistinguishable by pulsed-field gel electrophoresis. ST382 appeared to be an emerging clone that began to diverge from its ancestor approximately 32 years before 2016. We estimated that there was 1.29 nucleotide substitution per genome (2.94 Mbp) per year for this clone.

## INTRODUCTION

Three notable multistate outbreaks linked to contaminated produce were reported between 2014 and 2016, including a 2014 outbreak linked to contaminated stone fruit (peaches, nectarines, plums, and pluots) (1, 2), a 2014 to 2015 outbreak linked to contaminated caramel apples (3), and a 2015 to 2016 outbreak linked to packaged leafy green salad (4). In the case of the caramel apple and leafy green salad outbreaks, case patients in Canada were also identified (3, 4). Recently, a clinical case in Australia was plausibly linked to the stone fruit outbreak (5).

The real-time Listeria project of the United States federal and state agencies, in which the Food and Drug Administration (FDA) and Department of Agriculture primarily sequence food and environmental isolates and Centers for Disease Control and Prevention (CDC) primarily sequence clinical isolates, enables different outbreak-associated isolates to be fully sequenced and stored at the National Center for Biotechnology Information (NCBI) (6, 7). NCBI generates a daily updated single nucleotide polymorphism (SNP)-based whole-genome sequencing (WGS) tree (http://www.ncbi.nlm.nih.gov/pathogens/isolates/), providing an initial signal of clustering, to be followed up by epidemiological investigation and additional WGS analyses (e.g., reference-based SNP analysis and whole-genome multilocus sequence typing [wgMLST]). The implementation of global epidemiological surveillance through GenomeTrakr and PulseNet enabled tracing the spread of the outbreak strains outside the United States (1, 3, 5, 6, 8).

Analysis by a 7-gene multilocus sequence typing (MLST) scheme revealed that the Listeria monocytogenes population is largely clonal; most strains were scattered into major clonal groups, designated clonal complexes (CCs) or singletons (9, 10). A clonal complex (CC) is defined as a group of isolates exhibiting sequence types (STs) differing by no more than one allele from at least one other ST in the group (9, 10). A singleton is a clonal group that has an ST that differs from all other existing STs by at least two alleles, meaning a singleton does not belong to any existing CCs (9, 10). This population structure was recently confirmed by two core genome MLST (cgMLST) studies, revealing that isolates within a typical clonal group were closely related (up to 150 and 167 allele differences out of 1,748 and 1,827 core genes, respectively). In contrast, isolates from different clonal groups of the same evolutionary lineage were much more distant (up to 1,400 and 1,500 allele differences out of 1,748 and 1,827 core genes, respectively), as illustrated by noticeably longer tree branches (i.e., indication of genetic diversity) between different clonal groups than those within the same clonal group in the phylogenetic trees generated by the two cgMLST schemes (11, 12). It has been proposed recently that the clonal groups of L. monocytogenes be redefined as sublineages using cgMLST (12). However, we still use the term clonal complex in this study. MLST analyses on a subset of isolates involved in the 3 produce-associated outbreaks indicated that those isolates belonged to an L. monocytogenes singleton that had sequence type 382 and was accordingly designated ST382 (11). Because singleton ST382 was associated with three outbreaks, it was also classified as an epidemic clone (11). These isolates were serotype 4b by antiserum-based serotyping and serotype IVb-v1, a 4b variant, by PCR serotyping (1). Another outbreak strain, exhibiting ST1 of clonal complex 1 (CC1), was associated with the caramel apple outbreak. One clinical isolate of the ST382 outbreak strain and one clinical isolate of the CC1 outbreak strain (see Table S1 in the supplemental material) came from a single case-patient associated with the caramel apple outbreak (3). In addition, both ST382 and CC1 strains were obtained from distribution chain whole apples manufactured in the implicated facility (see Table S1), confirming that both strains were part of the caramel apple outbreak. Only ST382 isolates were associated with the stone fruit and leafy green salad outbreaks.

The ST382 isolates from these outbreaks are notable because, in contrast to other clonal groups that exhibited multiple pulsed-field gel electrophoresis (PFGE) profiles (13, 14), the isolates from the three outbreaks had one PFGE profile (GX6A16.0135/GX6A12.0349). It is also the least diverged epidemic clone identified to date (11). In addition, singleton ST382 isolates were not found in 2 large-scale MLST studies that sampled over 8,000 L. monocytogenes isolates (9, 15), indicating that ST382 might be an emerging clone. Identifying genetic variations among different ST382 strains will help us better understand the diversity and evolution of this emerging clone. cgMLST schemes could be applied to identify clonal groups of L. monocytogenes comprising different strains from multiple outbreaks/incidents (11). The reference-based SNP method has been used to cluster isolates from a single listeriosis outbreak (1), but has not been well established for clustering isolates in the same clonal group of L. monocytogenes but from different outbreaks/incidents. Our objectives here were (i) to determine whether the SNP-based WGS analysis can simultaneously detect ST382 and differentiate among ST382 strains from different outbreaks and epidemiologically unrelated sources, (ii) to identify genomic variations differentiating the 3 outbreak strains and the genomic diversity among isolates of each outbreak strain, and (iii) to determine at what point ST382 began diverging from its common ancestor.

## RESULTS

**FIG 1:**
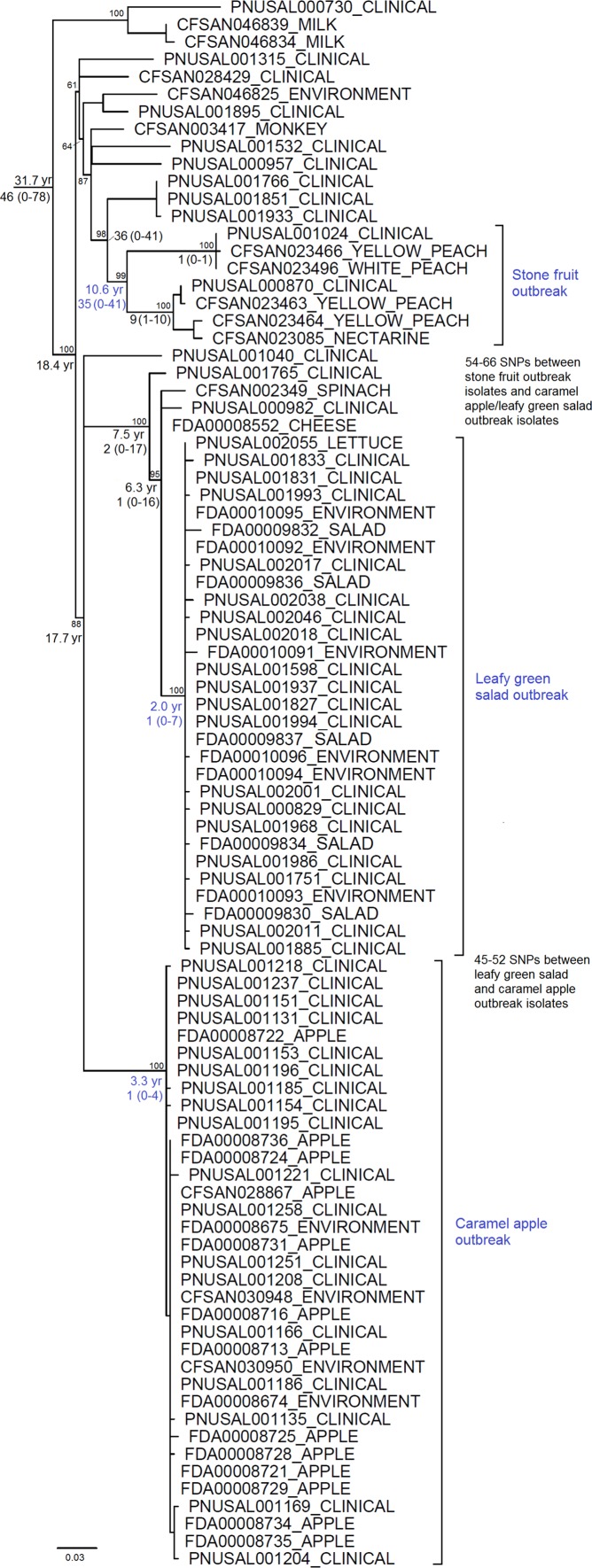
Maximum likelihood tree of all ST382 isolates constructed using GARLI on SNPs identified by the CFSAN SNP pipeline. The tree is rooted at the midpoint. The median, minimal, and maximal numbers of SNP differences among isolates within major clades are shown, with the minima and maxima in parentheses. The divergence time (years) is listed by the root of each major clade. The bootstrap values of major nodes are listed near each node. The divergence time (years) prior to March 2016 was generated by BEAST without CFSAN003417 because its isolation date was not available. BEAST analysis generated a tree with identical phylogeny for isolates associated with the three outbreaks, and nearly identical phylogeny for other isolates; thus, the BEAST tree is not shown.

All CC1 isolates from food, environment, and clinical cases associated with the caramel apple outbreak differed from each other by 0 to 9 (median, 2) SNPs, calculated without counting gaps. This indicated that these might be one strain, given the WGS diversity of isolates observed in previous outbreaks, as discussed below. Using CC1 isolates for comparison demonstrated that our SNP-based analysis clustered all ST382 isolates together and separated them from CC1 isolates, even though they both belonged to serotype 4b (see Fig. S1 in the supplemental material). A comparison across all ST382 isolates analyzed in this study, using one of the ST382 isolates as the reference, revealed 342 polymorphic loci and that any pair of isolates differed by up to 78 (median, 46) SNPs. The ST382 isolates associated with each of the 3 outbreaks, including those from food, packing/processing environments, and clinical cases, were grouped into three distinct clusters (Fig. 1). The pairwise SNP differences among ST382 isolates associated with the caramel apple and leafy green salad outbreaks ranged from 0 to 4 (median, 1) and from 0 to 7 (median, 1), respectively. Thus, the ST382 isolates associated with these two outbreaks may each represent a strain. Isolates associated with the stone fruit outbreak differed by 0 to 41 SNPs. Forty one SNPs are larger than SNP differences (31 to 40 SNPs) between isolates from the stone fruit outbreak and their nearest neighbors (PNUSAL001766, PNUSAL001851, and PNUSAL001933) that were epidemiologically unrelated to the outbreak, but WGS still placed the stone fruit outbreak isolates into one distinct cluster. The leafy green salad outbreak strain was genetically closer to the caramel apple outbreak strain than to the stone fruit outbreak strain (Fig. 1).

Analyzing multiple isolates of each outbreak strain enabled us to identify SNPs that specifically distinguished the three outbreak strains. Twenty SNPs (12 nonsynonymous, 7 synonymous, and 1 intergenic) were specific to the ST382 caramel apple outbreak strain. Five SNPs (4 nonsynonymous and 1 synonymous) were specific to the stone fruit outbreak strain. Six SNPs (4 nonsynonymous, 1 synonymous, and 1 intergenic) were specific to the salad outbreak strain (Table 1). Three-way genomic comparisons of the 3 outbreak strains, each represented by multiple isolates, did not reveal definitive evidence of unique coding regions among the 3 outbreak strains. The mapping of other ST382 strains, each represented by one or two isolates, against the complete genome of CFSAN023463 revealed 2 to 12 coding regions missing in other ST382 draft genomes, but those could be due to incomplete coverage of draft sequencing and should not serve as definitive evidence of missing coding regions. PHASTER identified a putative prophage between positions 141751 and 152479 of CFSAN023463, although it was listed as questionable by the software. This putative prophage was present in all ST382 strains, containing only 2 polymorphic loci. All CC1 isolates associated with the caramel apple outbreak had the same prophage sequence, which was identical to the 10.7 kbp monocin between positions 132409 (start of gene LMOf2365_0131) and 143137 (end of gene LMOf2365_0147) of a CC1 strain (F2365, updated annotation NC_002973) associated with a 1985 U.S. cheese outbreak (16). It differed from the prophage of CFSAN023463 by 8 SNPs, indicating limited prophage divergence. The ST382 isolates possessed the newly proposed Listeria pathogenicity island 4 (LIPI-4), which was initially identified in CC4 (12). The ST382 isolates did not have premature stop codons in *inlA*.

**TABLE 1:** SNPs specific to the entire set of isolates from the stone fruit, caramel apple, and leafy green salad outbreaks

Position^*a*^	Nucleotide			Amino acid		Putative protein identifier and function^*a*^
	Reference	Variant	Syn^*b*^	Reference	Variant	
Caramel apple outbreak						
129234	C	T	No	S	L	KO21_RS00690, chitinase
596119	C	A	Yes	—^*c*^	—	KO21_RS02920, Zn-dependent hydrolase
740311	G	A	Yes	—	—	KO21_RS03645, flagellar biosynthesis protein FliP
771828	C	T	No	T	I	KO21_RS03820, flagellar basal body rod protein FlgC
891117	T	G	No	Y	D	KO21_RS04410, hypothetical protein
1010170	T	C	—	—	—	intergenic
1206429	C	T	No	T	I	KO21_RS06050, microcompartment protein PduM
1208160	A	G	No	N	S	KO21_RS06065, aldehyde dehydrogenase
1328766	C	A	No	T	K	KO21_RS06690, DNA topoisomerase IV subunit A
1351776	C	T	No	D	N	KO21_RS06800, helicase SNF2
1517586	A	T	No	I	N	KO21_RS07605, molecular chaperone DnaJ
1695243	G	A	Yes	—	—	KO21_RS08430, LD-carboxypeptidase
1706380	C	T	No	R	H	KO21_RS08465, exonuclease
1911730	T	G	Yes	—	—	KO21_RS09510, primosomal protein N
1934615	C	T	No	G	E	KO21_RS09615, NCS2 family permease
2030550	G	A	Yes	—	—	KO21_RS10100, peptidoglycan-binding protein LysM
2159359	G	A	Yes	—	—	KO21_RS10665, hypothetical protein
2211332	G	A	No	P	S	KO21_RS10950, serine/threonine protein phosphatase
2267308	A	G	Yes	—	—	KO21_RS11235, alkyl sulfatase
2456769	A	G	No	K	R	KO21_RS12190, membrane protein
Packaged leafy green salad outbreak						
493309	C	A	No	A	E	KO21_RS02460, wall-associated RHS family protein
810535	A	T	No	E	V	KO21_RS04025, glyoxalase
1014483	G	A	No	T	M	KO21_RS05020, hypothetical protein
1271026	T	C	Yes	—	—	KO21_RS06390, DNA polymerase/3′–5′ exonuclease PolX
1476887	C	T	—	—	—	Intergenic
2092881	A	C	No	F	V	KO21_RS10390, NADP-dependent aryl-alcohol dehydrogenase
Stone fruit outbreak						
760144	T	A	No	L	M	KO21_RS03750, flagellar hook protein FlgE
1645676	T	G	No	K	T	KO21_RS08195, N-acetylglutamate synthase
2427859	A	G	No	D	G	KO21_RS11980, glutamate/gamma-aminobutyrate antiporter
2647562	T	C	No	Y	C	KO21_RS13120, amidase
2734870	C	T	Yes	—	—	KO21_RS13635, PTS sugar transporter subunit IIA

a Information is based on position in the reference genome of CFSAN023463 (GenBank accession no. NZ_CP012021.1 ).

b Syn, synonymous change.

c —, not applicable.

BEAST analysis, based on a current date of 24 March 2016, revealed that ST382 started to diverge 31.7 years ago (95% credible interval [CI], 24.5 to 39.9). The isolates associated with the stone fruit, leafy green salad, and caramel apple outbreaks began to diverge 10.6 (95% CI, 7.7 to 13.9), 2.0 (95% CI, 1.3 to 2.8), and 3.3 (95% CI, 2.3 to 4.5) years ago, respectively, which means they began diverging approximately 8.9, 1.7, and 2.0 years, respectively, before the recognition of each outbreak. The ancestor of the leafy green salad and caramel apple outbreak strains began to diverge 17.7 (95% CI, 12.9 to 22.7) years ago. The ancestor of all three outbreak strains began to diverge 18.4 (95% CI, 14.8 to 23.2) years ago. The geometric mean estimate of the number of nucleotide substitutions per genome per year was 1.29 (95% CI, 0.96 to 1.67).

## DISCUSSION

### SNP-based WGS analysis can be used for identifying ST382 and differentiating among the 3 outbreak strains and other epidemiologically unrelated strains.

Using SNPs identified by the FDA Center for Food Safety and Nutrition (CFSAN) SNP pipeline enabled WGS to differentiate ST382 isolates as a group from CC1 isolates, both belonging to serotype 4b. The reference-based SNP analysis also distinguished isolates from different outbreaks and other epidemiologically unrelated sources, demonstrating the power of WGS, since 84 of the ST382 strains with PFGE information available in this study, except FDA00008552, exhibited the same PFGE profile. For each of the 3 multistate outbreaks, food, environmental, and clinical isolates clustered together. A clinical isolate, PNUSAL001765, was included in the salad outbreak investigation, but it was outside the WGS cluster of the outbreak isolates. A relatively large number of isolates from each outbreak was analyzed to identify the diversity among isolates of each outbreak strain and to identify SNPs highly specific to each outbreak, which might be useful for future functional genomics. Reference-based methods require reference genomes and target genomes to be genetically close for maximum resolution and SNP calling accuracy (17), which is why we used an ST382 outbreak strain as the reference. Isolates in this clone differed by up to 78 SNPs, indicating that they were relatively closely related compared with isolates within other clonal groups, e.g., clonal complex 5 isolates from different outbreaks/incidents that differed by more than 250 SNPs (1). In the future, the SNP-based method could be evaluated using other clonal groups of L. monocytogenes.

Whole-genome level diversity among isolates associated with a common-source outbreak was a topic of many previous studies, which identified such diversity to be up to 3 (18), 10 (19), 20 (20), and 28 (21) SNPs, although we need to keep in mind that different studies might target slightly different portions of the genome and employ different bioinformatics tools or parameters to identify SNPs. This information can be useful in assessing whether a WGS clade of epidemiologically related isolates represents one strain. For example, we believe that the CC1 isolates associated with the caramel apple outbreak (0 to 9 SNPs), ST382 isolates associated with the caramel apple outbreak (0 to 4 SNPs), and ST382 isolates associated with the packaged salad outbreak (0 to 7 SNPs) should each represent one strain. Whether or not we should really define the stone fruit isolates as one strain is an intriguing question. The stone fruit cluster (up to 41 SNPs) consisted of two clades, each containing isolates differing by up to 10 SNPs; however, no other ST382 isolates fell between these two clades. Therefore, we caution that more WGS analyses are needed before we establish a cutoff value of genetic diversity to define bacterial strains. Notably, the maximum SNP difference (41 SNPs) between isolates associated with the stone fruit outbreak was larger than the SNP differences (up to 40 SNPs) between the stone fruit cluster and its nearest neighbors; however, they were clearly separated by the maximum likelihood phylogeny based on the whole-genome SNP matrix, well supported by bootstrap values (Fig. 1). Similarly, the packaged salad outbreak isolates and the 3 most genetically close, epidemiologically unrelated isolates (PNUSAL000982, FDA00008552, and CFSAN002349) differed by only 7 to 16 SNPs, smaller than the diversity of isolates associated with some other outbreaks (20, 21). This indicates that we should not rely solely on some threshold of WGS diversity (i.e., number of SNPs) to delineate an outbreak, rather, we should always interpret isolate diversity in the context of a given WGS phylogeny and epidemiological evidence.

ST382 was first recognized by its association with outbreaks under discussion in this study. However, available data from GenomeTrakr and PulseNet showed that ST382 strains may have been distributed across the United States for a few years (see Table S1 in the supplemental material). Specifically, the isolates analyzed in this study were from food sources from as early as 2002, clinical sources from as early as 2003, and an animal source with no isolation date available. The presence of ST382 isolates outside the United States, not associated with the outbreaks under discussion in this study, has not been reported to date. BEAST analysis suggested that ST382 began diverging from its ancestor 31.7 years ago, indicating that it is a relatively newly diverged clone, which could explain its limited divergence. In contrast, other major epidemic clones have existed for 50 to 150 years as of 2016 (12). Notably, CC1 was estimated to have existed 140 years ago, and it was observed in three continents (Europe, North America, and Asia) at least 57 years ago and in two additional continents at least 51 years ago (9). This indicates that the global distribution of most epidemic clones might not be very recent epidemiological events. However, the term epidemic clone is still informative by indicating the potential of a clonal group to be repeatedly involved in listeriosis outbreaks (22). The facilities implicated in the stone fruit and caramel apple outbreaks were located approximately 130 km from each other in the same state. However, they were well separated by WGS, and the divergence from and initial transmission of their ancestor were not recent (estimated to be 18.4 years ago). The caramel apple outbreak strain was actually genetically closer to the leafy green salad outbreak strain that contaminated a facility approximately 3,500 km away than to the stone fruit outbreak strain (Fig. 1). Nonetheless, the contamination of food production environments by Listeria may be a complicated scenario, possibly due to persistence and/or repeated introduction, especially considering the clonal spread of L. monocytogenes could occur rather rapidly (23). In addition, the evolutionary rates calculated by BEAST were based on WGS of the entire collection of ST382 isolates, and we cannot exclude the possibility that a specific clade of isolates, e.g., those associated with an outbreak, evolved faster or slower than average due to the selective pressures they endured in the specific produce growing and/or packing/processing environments. The available information showed that the environmental isolates associated with the caramel apple outbreak, analyzed in this study, were from both food contact and nonfood contact surfaces. Such detailed information was not available for those associated with the stone fruit and leafy green salad outbreaks, and no samples from orchards/farms were available for analysis. Therefore, extensive and longitude surveillance work is needed to fully elucidate the evolution and transmission events of ST382, and L. monocytogenes in general, in the various food production environments.

Even though our analysis targeted the entire genome, we did not find evidence that significant portions of the coding regions were missing from different strains, at least in genomic regions covered by draft sequencing. Thus, the whole genome we analyzed is essentially the core genome of ST382. The geometric mean nucleotide substitution rate was 1.29 (95% CI, 0.96 to 1.67) nucleotides per genome (2.94 Mbp whole genome) per year, which is in line with the average mutation rate (0.41 substitutions per 1.58 Mbp core genome per year) estimated for CC1 (12).

The implementation of WGS for global epidemiological surveillance of listeriosis outbreaks contributed to the identification of cross-country transmission of the ST382 outbreak strains. Clinical cases in Canada were linked to the caramel apple and leafy green salad outbreaks (3, 8). WGS of the Canadian clinical isolates were not publically available, and thus analysis of those isolates is not presented here. The clinical case in Australia linked to the 2014 stone fruit outbreak was initially treated as a sporadic case. The WGS of this clinical isolate in Australia was submitted to GenomeTrakr in December 2015 and clustered with the U.S. outbreak-associated isolates by the NCBI daily updated SNP tree. The clustering was subsequently confirmed by the reference-based SNP analysis (5). Thus, WGS demonstrated its potential for tracking the international spread of L. monocytogenes. A whole-genome MLST method was used to assist in investigating a multinational outbreak in Germany and Austria (24). In two retrospective studies, a core genome MLST method identified the transmission of an outbreak strain from Italy to the United States (11), and another core genome MLST scheme revealed evidence of international transmission events, although no epidemiological links were identified among the isolates of those international clusters due to the retrospective nature of the study (12).

### ST382 and CC1 isolates had a conserved 10.7 kbp putative incomplete prophage or monocin.

Prophage regions in L. monocytogenes could diverge rapidly and offer genetic markers for differentiating closely related isolates with limited or no diversity in the core genome (19). However, only 2 polymorphic loci identified by WGS were in the putative prophage/monocin among the ST382 isolates. The 8 SNPs between the CC1 genomes and the ST382 reference genome (CFSAN023463) were much smaller than the differences among epidemiologically unrelated strains in other prophages (25, 26), indicating that this putative prophage/monocin was highly conserved between both clonal groups. The putative prophage in CFSAN023463 was previously identified by PHAST to be 22.9 kbp (1), inclusive of the prophage identified by PHASTER in this study. We believe PHASTER was more accurate in identifying the start and end of this prophage because its result was consistent with the previous identification of this prophage/monocin in F2365 (16).

### Contamination of produce by L. monocytogenes represents an emerging public health concern.

Elderly patients were involved in all three outbreaks. The stone fruit and caramel apples were both novel vehicles of listeriosis outbreaks. In the caramel apple outbreak, most case-patients consumed caramel apples, but L. monocytogenes was isolated from whole apples produced in the implicated apple manufacturing facility. Whole apples do not support the growth of L. monocytogenes (27, 28), but the production of caramel apples requires the insertion of a stick into the fruit, which may have facilitated the transfer of juice from the interior to the surface, thereby creating a microenvironment at the apple-caramel interface that enabled L. monocytogenes to multiply (27). Caramel apples are often given to children, which could partially explain why this outbreak involved 3 otherwise healthy children, aged between 5 and 15 years, a typically low-risk group (3). These 3 children yielded 3 ST382 isolates and 1 CC1 isolate. One otherwise healthy child in the packaged leafy green salad outbreak developed meningitis (4). The pH of the stone fruit pulp is generally below 4.0 (29), which does not favor the growth of L. monocytogenes, and L. monocytogenes did not grow on the surface of stone fruit (30). Leafy greens support the growth of L. monocytogenes (31, 32). However, this packaged leafy green salad outbreak represented the first reported listeriosis outbreak linked to leafy greens since 1981, which may be partially due to improved outbreak detection. Prior to 1981, listeriosis outbreaks associated with leafy greens included the 1979 Boston raw vegetable (celery, tomato, and lettuce) outbreak and the 1981 Canada coleslaw outbreak. However, the source tracking for these outbreaks was mostly based on epidemiological evidence, because high resolution subtyping techniques were not available (28). More studies are needed to understand whether certain L. monocytogenes isolates are uniquely adapted to produce and produce-growing/packing/processing environments.

### Conclusions.

SNP-based WGS analysis simultaneously detected ST382 and differentiated among ST382 isolates from different outbreaks and other epidemiologically unrelated sources. WGS analysis enabled an inference of the evolutionary history of ST382.

## MATERIALS AND METHODS

### WGS data.

From the GenomeTrakr database (http://www.ncbi.nlm.nih.gov/bioproject/183844), we downloaded WGS raw reads from multiple U.S. clinical, food, and environmental isolates associated with the 3 outbreaks linked to contaminated stone fruit, caramel apples, and packaged leafy green salad, which were analyzed by WGS SNP analysis and wgMLST during the investigations of each outbreak (1–4). The isolates associated with the caramel apple outbreak were from whole apples in the distribution chain, one sample of the caramel apple processing environment, food and nonfood contact surfaces of the whole apple packing/processing environment, and clinical cases. The isolates associated with the leafy green salad outbreak were from clinical cases and salad and its processing environment. The isolates associated with the stone fruit outbreak were from clinical cases and stone fruits, a subset that represented the diversity of the entire set of outbreak-associated isolates (1). A second set of ST382 isolates, not associated with the 3 outbreaks, was identified through GenomeTrakr and PulseNet based on the availability of WGS and metadata (see Table S1 in the supplemental material). This second set of isolates was from food, environment, and clinical cases and an animal source. We performed *in silico* MLST analysis on all isolates using tools in the BIGSdb-*Lm* database (http://bigsdb.pasteur.fr/listeria/listeria.html) to confirm that they all belonged to singleton ST382.

### Identification of SNPs.

The SNPs were identified using default settings of the FDA Center for Food Safety and Applied Nutrition (CFSAN) SNP pipeline, version 0.7.0, targeting the entire genome, including coding and noncoding regions from core and accessory genomes (33, 34). To demonstrate how the SNPs identified by the CFSAN SNP pipeline could be used to distinguish ST382 from isolates of the same serotype but a different clonal group, we first used CC1 isolates (see Table S1) associated with the caramel apple outbreak for comparison. We then performed the SNP analysis without CC1 isolates for precise identification of SNPs, because accurate SNP calling by reference-based methods may be affected by ascertainment bias when these methods are applied to slightly more diverse isolates (17, 35). The complete genome of CFSAN023463 (GenBank accession no. NZ_CP012021.1) from the stone fruit outbreak was used as the reference. Briefly, raw reads from each isolate were mapped to the reference genome using default settings of Bowtie 2 version 2.2.9 (36). The BAM file was sorted using SAMtools version 1.3.1 (37), and a pileup file for each isolate was produced. These files were then processed using VarScan2 version 2.3.9 (38) to identify high-quality variant sites using the mpileup2snp option. A Python script was used to parse the.vcf files and construct an initial SNP matrix. To remove regions that may have recombination and/or low-quality sequencing/mapping and/or be associated with repetitive elements from the final SNP matrix, 6 high-density variant regions (≥3 variant sites in ≤1,000 bp of any one genome) containing 79 variant sites were subsequently removed by a filter. The excluded regions were 802 bp (containing 12 variant sites), 592 bp (14 sites), 259 bp (31 sites), 15 bp (4 sites), 196 bp (15 sites), and 10 bp (3 sites), all in leucine-rich repeat or tandem repeat-containing regions. To identify any major coding regions specific to each outbreak strain, two additional raw reads mappings were performed. The reference genomes were FDA00008716 (286× coverage, from the caramel apple outbreak) and FDA00009837 (200× coverage, from the leafy green salad outbreak), both assembled using CLC Genomics WorkBench 9.0.1 (Qiagen, Aarhus, Denmark). Coding regions were filtered using default settings of Ridom SeqSphere^+^ version 3.4 (Ridom GmbH, Germany) (39). A separate SNP analysis was performed on only the CC1 isolates associated with the caramel apple outbreak, using FDA00008730 (GenBank accession no. NZ_LYXY00000000.1) as the reference, to precisely determine the number of SNPs among the isolates.

### Evolutionary analysis.

The genetic algorithm for rapid likelihood inference (GARLI) (40) was used to infer a phylogeny based on the SNP matrix identified by the SNP pipeline. The default parameters of GARLI were used. The best tree was chosen among 100 replicates of the nonbootstrapped data set, and topological support was estimated from 1,000 bootstrap replicates. To further understand the evolutionary history of ST382 isolates, we took advantage of the heterochronous sampling of individuals and estimated divergence dates among these isolates using Bayesian evolutionary analysis by sampling trees (BEAST) version 1.8.0 (41), which estimates branch lengths in years. The HKY model of substitution (42) was used along with a coalescent model assuming a constant population size. Analyses were performed under both a strict molecular clock and a relaxed lognormal clock; uniform priors were used for clock.rate and ucld.mean, respectively. Based on the Akaike's information criterion through Markov chain Monte Carlo, which has been shown to be more robust than the harmonic mean method (43), the strict molecular clock was the better fit. In addition, there was a low level of diversity segregating among isolates, and they were sampled over a small temporal scale, both of which suggest a strict clock is the most appropriate model. Thus, results under the strict molecular clock model are presented here. This generated a phylogeny almost identical to that produced by GARLI. The analysis was run for 10^8^ generations, sampling every 10^4^ generation; a 10% burnin was used to estimate the best-fitting topology and divergence time. The effective sample size values were also monitored to ensure sufficient mixing and convergence. The current date was 24 March 2016, which was the most recent isolation date of all the isolates in this study. If the collection date of an isolate was only identified to a month, the first day of that month was assumed to be the collection date. If the collection date was only identified to a year, the first day of that year was assumed to be the collection date. CFSAN003417 did not have the isolation date available and was not included in the BEAST analysis.

### Prophage, Listeria pathogenicity island 4 (LIPI-4) and *inlA* analysis.

PHASTER (44) was used to identify putative prophages in the complete genome of CFSAN023463. The presence and divergence of this prophage in other ST382 genomes were determined using BLAST. The presence and divergence of this prophage in the CC1 isolates associated with the caramel apple outbreak were also determined. The presence/absence of a newly proposed Listeria pathogenicity island 4 (LIPI-4) (12), and the premature stop codons in internalin A (*inlA*) were determined using BLAST.

## Supplementary Material

Supplemental material
