# Supplementary material for: Singleton Sequence Type 382, an Emerging Clonal Group of Listeria monocytogenes Associated with Three Multistate Outbreaks Linked to Contaminated Stone Fruit, Caramel Apples, and Leafy Green Salad
Source: J Clin Microbiol. 2017 Feb 22;55(3):931–41. doi: 10.1128/JCM.02140-16 (PMC5328462; doi:10.1128/JCM.02140-16)
Supplement: Supplemental material [file supp_55_3_931__index.html]

Singleton Sequence Type 382, an Emerging Clonal Group of Listeria monocytogenes Associated with Three Multistate Outbreaks Linked to Contaminated Stone Fruit, Caramel Apples, and Leafy Green Salad — Supplemental material 

# Singleton Sequence Type 382, an Emerging Clonal Group of Listeria monocytogenes Associated with Three Multistate Outbreaks Linked to Contaminated Stone Fruit, Caramel Apples, and Leafy Green Salad

## Supplemental material

- Supplemental file 1 -

  Fig. S1 (Phylogenetic analysis of CC1 isolates associated with the caramel apple outbreak and all ST382 isolates, with CFSAN023463 as the reference)

  PDF, 4.4M
- Supplemental file 2 -

  Table S1 (Isolates from food, environmental, and clinical sources analyzed in this study)

  XLSX, 21K
