# Supplementary material for: Singleton Sequence Type 382, an Emerging Clonal Group of Listeria monocytogenes Associated with Three Multistate Outbreaks Linked to Contaminated Stone Fruit, Caramel Apples, and Leafy Green Salad
Source: J Clin Microbiol. 2017 Feb 22;55(3):931–41. doi: 10.1128/JCM.02140-16 (PMC5328462; doi:10.1128/JCM.02140-16)
Supplement: Supplemental material [file JCM.02140-16_zjm999095402s1.pdf]

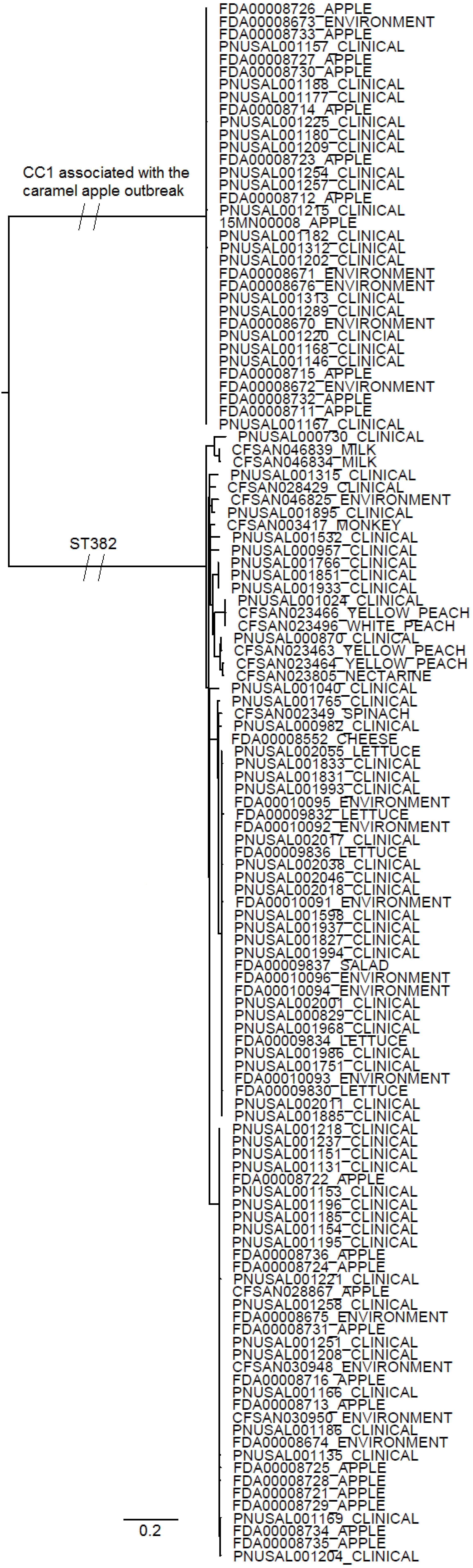

Figure S1. Phylogenetic analysis of CC1 isolates associated with the caramel apple outbreak and all ST382 isolates with CFSAN023463 as the reference. The tree uses midpoint rooting.
